# Synthetic Lethal Screen Identifies NF-κB as a Target for Combination Therapy with Topotecan for patients with Neuroblastoma

**DOI:** 10.1186/1471-2407-12-101

**Published:** 2012-03-21

**Authors:** Patricia S Tsang, Adam T Cheuk, Qing-Rong Chen, Young K Song, Thomas C Badgett, Jun S Wei, Javed Khan

**Affiliations:** 1 Oncogenomics Section, Pediatric Oncology Branch, Center for Cancer Research, National Cancer Institute, Bethesda, MD 20892, USA

## Abstract

**Background:**

Despite aggressive multimodal treatments the overall survival of patients with high-risk neuroblastoma remains poor. The aim of this study was to identify novel combination chemotherapy to improve survival rate in patients with high-risk neuroblastoma.

**Methods:**

We took a synthetic lethal approach using a siRNA library targeting 418 apoptosis-related genes and identified genes and pathways whose inhibition synergized with topotecan. Microarray analyses of cells treated with topotecan were performed to identify if the same genes or pathways were altered by the drug. An inhibitor of this pathway was used in combination with topotecan to confirm synergism by *in vitro *and *in vivo *studies.

**Results:**

We found that there were nine genes whose suppression synergized with topotecan to enhance cell death, and the NF-κB signaling pathway was significantly enriched. Microarray analysis of cells treated with topotecan revealed a significant enrichment of NF-κB target genes among the differentially altered genes, suggesting that NF-κB pathway was activated in the treated cells. Combination of topotecan and known NF-κB inhibitors (NSC 676914 or bortezomib) significantly reduced cell growth and induced caspase 3 activity *in vitro*. Furthermore, in a neuroblastoma xenograft mouse model, combined treatment of topotecan and bortezomib significantly delayed tumor formation compared to single-drug treatments.

**Conclusions:**

Synthetic lethal screening provides a rational approach for selecting drugs for use in combination therapy and warrants clinical evaluation of the efficacy of the combination of topotecan and bortezomib or other NF-κB inhibitors in patients with high risk neuroblastoma.

## Background

Neuroblastoma is the most common extra-cranial solid tumor in childhood, accounting for 7-10% of childhood cancers [1]. Based on age, staging, *MYCN *amplification status, histology, and DNA ploidy, neuroblastoma is classified into low, intermediate and high risk groups [2,3]. At present, high risk neuroblastoma is treated with high dose chemotherapy, surgery, autologous stem cell transplantation, radiation, immune and differentiating therapy. Currently used chemotherapeutic agents in standard and salvage regimens include toposisomerase I and II inhibitors, topotecan, etoposide, irinotecan and doxorubicin; alkylating agents, cisplatin, carboplatin, melphalan and cyclophosphamide and the microtubule inhibitor vincristine [4,5]. The differentiating agent 13-cis-retinoic acid is also administered during the maintenance period post chemotherapy. Recent clinical trials have shown that the combination of anti-GD2 antibodies and immunocytokines significantly increase the survival of patients with high risk neuroblastoma [6,7]. Despite these aggressive combined multimodal treatments the survival rate for these high risk neuroblastoma patients remains less than 50%.

Topoisomerase inhibitors are currently a mainstay of many salvage regimens for neuroblastoma and are being evaluated as up-front therapy in an ongoing trial [8-11]. They function by perturbing the cellular machinery responsible for maintaining DNA structure during transcription and replication. Topotecan is an inhibitor for the enzyme topoisomerase-I which is involved in the replication and repair of nuclear DNA. As DNA is replicated in dividing cells, topoisomerase-I binds to super-coiled DNA causing single-stranded breaks. As a result, topoisomerase-I relieves the torsional stresses that are introduced into DNA ahead of the replication complex or moving replication fork. Topotecan inhibits topoisomerase-I by stabilizing the covalent complex of enzyme and strand-cleaved DNA, which is an intermediate of the catalytic mechanism, thereby inducing breaks in the protein-associated DNA single-strands, resulting in cell death [12]. This agent is currently used for the treatment of many cancers including metastatic ovarian cancer and platinum-sensitive relapsed small-cell lung cancer [13], recurrent or persistent cervical cancer [14], and neuroblastoma [15]. In addition, topotecan is being evaluated in pediatric cancer patients for treating leukemia, lymphoma, Ewing's sarcoma, rhabdomyosarcomas and gliomas (http://www.clinicaltrials.gov). However, the primary dose-limiting toxicity of topotecan is myelosuppression, restricting its use at high doses. Therefore, identification of other chemotherapeutic agents synergizing with topotecan may potentially maintain or increase efficacy while limiting toxicity.

In this study, we performed a loss-of-function synthetic lethal siRNA screening of 418 apoptosis related genes with and without topotecan to identify genes or pathways whose inhibition synergized with topotecan to enhance growth suppression or apoptosis in neuroblastoma. The goal of the study was to identify drugs that would potentially be synergistic when used in combination with topotecan to inhibit the growth of neuroblastoma.

## Methods

### Cell lines and culture conditions

The neuroblastoma cell lines SK-N-AS and SH-SY5Y were maintained in RPMI-1640; and NB-1691 was maintained in DMEM, both supplemented with 10% FBS, 1% penicillin/streptomycin (P/S) and 1% L-glutamine (all from Quality Biological Inc., Gaithersburg, MD) at 37°C. To ensure consistency, a batch of cells was expanded, aliquoted and stored in liquid nitrogen prior to the screening. In each experiment, a vial of cells was defrosted and passaged 1:4 when 70% confluency was reached. Cells between passages 3 and 7 were used for all experiments.

### Reagents

Topotecan hydrocholoride (Hycamtin; GlaxoSmithKline, Philadelphia, PA) and Bortezomib (Velcade; Millenium Pharmaceuticals, Cambridge, MA) were reconstituted and stored according to the manufacturers' instructions. NSC 676914 was obtained from the Developmental Therapeutics Program, Division of Cancer Treatment and Diagnostics, NCI/NIH.

### High throughput siRNA screening

A set of synthetic siRNAs targeting 418 genes related to the apoptotic pathway (Qiagen Apoptosis Set V.1; Qiagen, Valencia, CA), with 2 siRNAs of different sequences per gene, was used for the first screen. For the second screen, 2 new siRNA pre-designed sequences were used (Qiagen). In the third confirmatory screen, one siRNA from each of the previous two screens was chosen. siRNAs were transfected at passage 4. Briefly, transfection reagent Dharmafect 1 (Dharmacon RNA Technologies, Lafayette, CO) was diluted in DCCR reagent medium at a ratio of 1:208 in volume. siRNA (20 nM) and 25 μL of the diluted transfection reagent were added to an individual well in a 96-well plate for complex formation with incubation for 20 min at RT. SK-N-AS cells were trypsinized, counted and resuspended in P/S free culture medium. 5000 cells were added to each individual well in 100 μL medium. The plate was incubated at RT for 30 min for cell attachment before being placed at 37°C for 24 h. Topotecan was then added to each well for additional 72 h incubation. Cell proliferation assay was performed at 96 h post siRNA transfection. The IC50 of topotecan for SK-N-AS cells at 24 h was 2 μM. In the 1st and 2nd screens, topotecan doses of 0, 1, 5 and 10 μM were used, whereas in the 3 rd screen, lower drug doses of topotecan at 0, 0.01, 0.1 and 1 μM were used to identify synergy. The criterion of hit selection for the enhancer genes was ≤0.8 fold cell growth compared to its own siRNA effect in the presence of topotecan. The final enhancer gene list was subjected to pathway analysis for identification of overrepresented genes within a target pathway. An inhibitor to the pathway was chosen and tested individually or in combination with topotecan *in vitro *and *in vivo*.

### Cell proliferation assay

Cell proliferation was measured using Cell Titer Glo proliferation assay (Promega Corporation, Madison, WI) or a real-time cell sensing system (RT-CES; ACEA Biosciences, Inc. San Diego, CA) according to the manufacturer's instruction. We performed cell proliferation assays at a volume of 35 μL per well for 10 min and was measured at 562 nm on a Tecan plate reader (Tecan Inc., Durham, NC). Each treatment was performed in triplicates, averaged and normalized using untreated cells. For real-time cell electronic sensing assays, cells treated with drugs alone or in combinations or transfected with siRNA were added in triplicates to a 96-well plate device compatible with the real-time cell electronic sensing analyzer (RT-CES; ACEA Biosciences, Inc. San Diego, CA). Cell growth was monitored hourly for indicated durations via calculation of "cell index" (normalized impedance) for each well.

### Quantitative RT-PCR

SK-N-AS cells were collected at 60 h post siRNA transfection. Total RNA was reverse transcribed using SuperScript II reverse transcriptase system (Life Technologies, Foster City, CA). The target regions were then pre-amplified using a standard PCR for 10 cycles. The pre-amplified cDNA was quantified by Taqman gene expression assay (Life Technologies) using Fluidigm digital array (South San Francisco, CA) according to the manufacturer's protocol. Fold expression was calculated using a comparative threshold cycle method (2^-Δ ΔCT^) [16].

### Pathway analysis

The pathway analysis was performed using MetaCore (http://www.genego.com/metacore.php, GeneGo Inc., St Joseph, MI). MetaCore is an integrated software suite for functional analysis of experimental data and it contains curated protein interaction networks on the basis of manually curated database of human protein-protein, protein-DNA, protein-RNA and protein-compound interactions. Metacore uses a hypergeometric model to determine the significance of enrichment. The enhancer genes from our experiment and the genes in the GeneGo maps from the MetaCore database were used to identify the enriched GeneGo pathway maps.

### Gene Set Enrichment Analysis

To investigate gene set enrichment, GSEA (http://www.broad.mit.edu/gsea/) was performed for genes, ranked by log_2 _ratio of gene expressions between topotecan-treated (1 μM and 10 μM) and untreated control SK-N-AS cells, using a weighted Kolmogorov-Smirnov-like statistics [17]. The gene set of NF-κB target genes used in GSEA were downloaded from http://bioinfo.lifl.fr/NF-KB.

### Western blotting

Cells were seeded on a 100 mm^2 ^culture dish for overnight and were treated with topotecan alone or with bortezomib. For topotecan alone effect, SK-N-AS were treated with 5 μM of topotecan for 0, 3, 6, or 24 h at 37°C. For the combined effect of bortezomib and topotecan, cells were treated with bortezomib at 0, 1 and 10 nM for 24 h, followed by the addition of topotecan at 5 μM for 6 h. Total cell lysate was collected with RIPA buffer containing 1% phosphatase inhibitor and 1% protease inhibitor (all from Thermo Fisher Scientific, Rockford, IL). Protein concentration was measured by BCA Protein Assay Kit (Thermo Fisher Scientific, Waltham, MA) and protein lysates (20 ug per lane) were resolved on 4-12% TRIS-gradient gel (Invitrogen Life Technologies, Carlsbad, CA) and were transferred to nitrocellulose membranes by iBlot blotting system (Invitrogen Life Technologies). The membranes were blocked with 5% non-fat dry milk in PBS with 0.1% Tween 20 (PBST) for 1 h at RT, followed by incubation with mouse monoclonal antibodies against total or phosphorylated IκB-α (Ser32/36), rabbit monoclonal antibodies against total or phosphorylated p65/RelA or rabbit monoclonal antibody against NFKB1 (all from Cell Signaling Technology, Danvers, MA) at 4°C overnight. Peroxidase-conjugated goat anti-mouse or anti-rabbit antibody was used as secondary antibodies (Santa Cruz Biotechnology, Santa Cruz, CA) for 1 h incubation at RT. Immunoreactive bands were visualized by ECL analysis system (GE Healthcare, Piscataway, NJ) and enhanced chemiluminescence. For loading control, the membranes were washed with Restore Western blot stripping buffer (Thermo Fisher Scientific) and probed with goat anti-human actin HRP conjugated antibody (Santa Cruz Biotechnology).

### *In vitro *drug combination

Neuroblastoma cells were trypsinized, counted and resuspended in P/S free culture medium. 5000 cells per well in 100 μL medium were seeded in 96-well white plates for overnight. Topotecan and NSC 676914 or bortezomib were added individually or in combination at various doses and the plates were incubated at 37°C for 24 h and 48 h respectively. Cell proliferation assay was performed as described above. Combination index was calculated using CompuSyn software (ComboSyn Inc., Paramus, NJ). Briefly, the combination index theorem was used to quantify synergy or antagonism for two drugs by the formula C.I. = (D)_1_/(D_x_)_1 _+ (D)_2_/(D_x_)_2_, where D1 and D2 are drug 1 and drug 2, and × is growth inhibition by X% [18].

### Apoptosis assay

The caspase-3 activity was measured using the PE Active Caspase-3 Apoptosis kit (BD Pharmingen, San Diego, CA). Briefly, SK-N-AS cells (untreated or treated with topotecan and bortezomib alone or in combination for 24 h) were trypsinized, fixed, and stained with PE rabbit anti-active caspase-3 antibody. Fluorescence intensity was measured by FACS Calibur and data were analyzed using CellQuest software (BD Biosciences, Franklin Lakes, NJ).

### *In vivo *xenograft model

All animal experiments have been reviewed and approved by the NIH Animal Care and User Committees. A minimal residual disease xenograft model in mice bearing neuroblastoma was established in 8-10 week-old female SCID Beige mice (Charles River Laboratories, Fredrick, MD). Briefly, five million SK-N-AS cells expressing luciferase (gift from Dr. Bryan Clary, Duke University Medical Center) were injected intravenously via the lateral tail vein into the mice. Tumors were allowed to grow for 7 d, and then mice were randomly assigned to cohorts treated with topotecan and bortezomib administered individually or in combination, or with saline solution (control mice). Bortezomib (0.6 mg/kg) was injected intraperitoneally three times a week for two weeks, rested for two weeks and repeated with another course of treatment. Topotecan (0.5 mg/kg) was injected intraperitoneally five times a week for two weeks, rested for two weeks, followed by another course of treatment. Body weight and general wellness of the mice were monitored, and tumor size was monitored by Xenogen IVIS 100 imaging system (Caliper Life Sciences Inc., Hopkinton, MA). The *in vivo *xenograft experiment was repeated, and results from two independent experiments were combined (n = 43).

### Statistical analysis

Non-parametric Mann-Whitney test was used to compare among various groups in cell growth assay. For relative luciferase intensity results from two independent *in vivo *experiments, we normalized the log2 transformed intensities from each experiment using median-centered method and then combined the results. *T*-test was used to compare the difference of two groups.

## Results

### Identification and validation of enhancer genes

Neuroblastoma cell line SK-N-AS was used to screen a siRNA library against 418 apoptosis related genes to identify genes whose inhibition can enhance the effect of topotecan in inhibiting neuroblastoma cell growth. Two siRNAs were initially used against each gene, either alone or in combination with various doses of topotecan. For the first screen, 64 out of the 418 siRNAs potentiated the growth inhibition induced by topotecan at any one of the three doses. To minimize possible off-target effects, the candidate genes were further screened with 2 new siRNA sequences in a second screen, and 18 hits continued to show efficacy. To validate theses hits, we performed a third confirmatory screen by using the two most effective siRNAs for each gene from the two screens and lower doses of topotecan. Nine hits including *BIRC4, CTSD, NFKB1, NOS2A, RIPK1, TGFB1, TNFRSF10A, TNFRSF25 *and *TNFRSF8*, were confirmed to potentiate the inhibition of cell growth by topotecan (Table 1).

**Table 1:** The common enhancer genes that potentiated the effect of topotecan from three screens

*Genes that enhance the effect of topotecan-induced cell death*						
BIRC4 *, **	BOK *	BBC3	CNTF	ICEBERG	PDCD2	TNFAIP1
CTSD *, **	CDKN2A *	BCL11B	DAP	IGF1	PIK3C2G	TNFAIP2
NFKB1 *, **	CDKN2D *	BCL2A1	DNASE1	IL10RB	PRDX5	TNFSF12
NOS2A *, **	F2R *	BIRC3	DOCK1	IL1B	PRF1	TNFSF5
RIPK1 *, **	MCL1 *	BNIP3	DUSP7	LTB	PTGS1	TP53
TGFB1 *, **	MMP9 *	CARD10	ERCC6	MAP2K6	RAD23B	TRAF1
TNFRSF10A *, **	STK3 *	CARD14	FAF1	MAP3K14	RB1	TRAF6
TNFRSF25 *, **	TNFRSF10D *	CASP9	GPX1	MYB	SFRP5	WDR3
TNFRSF8 *, **	TP53BP2 *	CLU	HSPE1	NOS3	TIA1	YWHAZ

Footnote: *genes present in the second screen (18); ** genes in all three screens (9)

### Inhibition of NF-κB pathway enhanced topotecan-mediated growth inhibition in neuroblastoma cells

**Figure 1:**
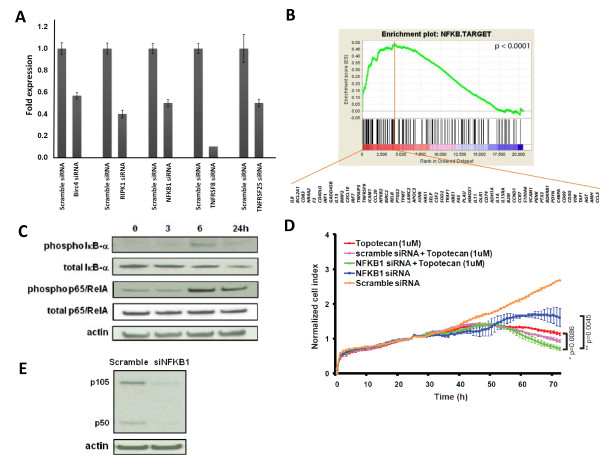
**Up-regulation of NF-κB pathway protects NB cells from tepotecan-mediated growth inhibition**. (**A**) Quantitative RT-PCR was performed on the 5 enhancer genes (Birc4, RIPK1, NfKB1, TNFRSF8 and TNFRSF25) to confirm target gene knockdown. Each gene expression is normalized with GAPDH and compared to siRNA control. Error bar represents the standard deviation of triplicate experiment. (**B) **GSEA analysis was performed on the ranked genes according to log_2 _ratio of gene expression between topotecan-treated and control SK-N-AS cells. The NF-κB target genes are significantly enriched in SK-N-AS cells treated with topotecan at 1 μM (p < 0.0001). The green curve shows the running sum of enrichment score (ES) for the ranked genes. Black vertical lines indicate gene hits in the NF-κB target gene set. The red vertical line marks the leading edge subset genes, and the NF-κB target genes within the leading edge subset genes are shown in their gene symbols. (**C**) Immunoblots showed activation of the NF-κB pathway by topotecan (5 μM) through phosphorylation and degradation of total IκB-α protein; and phosphorylation of p65/RelA in a time dependent manner. (**D**) Knockdown of *NFKB1 *potentiated the inhibitory effect of topotecan on cell growth when compared to topotecan alone (p = 0.0086), or *NFKB1 *siRNA alone (p = 0.0045) on a real-time cell electronic sensing system. (**E**) Immunoblots confirmed knockdown of NFKB1 protein (p105/p50 complex) of SK-N-AS cells transfected with *NFKB1 *siRNA for 72 hrs compared to scramble siRNA

We next explored if there were any signaling pathways associated with these nine hits using MetaCore (see Methods). The top of the list was anti-apoptotic TNFs/NF-κB/IAP pathway (p = 6.03E-06; Table 2). Out of the total 27 curated genes in this pathway, five of them were among the nine hits, including *BIRC4, NFKB1, RIPK1, TNFRSF25 *and *TNFRSF8*. The knockdown on these five target genes by siRNAs was confirmed using Taqman assays (Figure 1A). Therefore we hypothesized that the NF-κB pathway might be activated as a response to topotecan in SK-N-AS cells. To test this hypothesis, we performed Gene Set Enrichment Analysis (GSEA) [17] on microarray gene expression data of SK-N-AS cells treated with topotecan to determine if there was enrichment for NF-κB target genes (http://bioinfo.lifl.fr/NF-KB/). We found that these NF-κB target genes were significantly up-regulated in the leading edge subset genes in the GSEA analysis in the topotecan-treated cells (p < 0.0001; Figure 1B). Because activation of the NF-κB pathway results in degradation of IκB-α (through phosphorylation) and increase of p65/RelA phosphorylation, we further examined the effect of topotecan on the NF-κB pathway using Western blotting for these two key components of the pathway. Indeed, the Western blotting showed decreased amount of total IκB-α and increased phosphorylation of p65/RelA after topotecan treatment, confirming activation of NF-κB by topotecan (Figure 1C). These results suggested that the NF-κB pathway was activated by topotecan possibly as a protective mechanism against topoisomerase-I inhibition resulting in single stranded DNA breaks. To verify if the up-regulation of NF-κB pathway was protective from topotecan-mediated growth inhibition, we specifically knocked down *NFKB1*, another key component in the pathway, by siRNA. Knocking down *NFKB1 *resulted in further inhibition of cell proliferation in topotecan-treated cells (p < 0.01, Figure 1D), and a Western blot confirmed the effective knockdown of NFKB1 by the siRNA at the protein level (Figure 1E). Taken together, our results indicated that combination of topotecan with a NF-κB inhibitor might be synergistic, and this synergistic effect provided a rational combination therapy against neuroblastoma.

**Table 2:** Pathway analysis of the 9 common enhancer genes (P < 0.01)

*Genego Maps*	*Number of genes in the map*	*Number of overlapped enhancer genes*	*p-value*
**Anti-apoptotic TNFs/NF-κB/IAP pathway**	27	5	6.03E-06
**Apoptotic TNF-family pathways**	41	4	8.67E-04
**HTR1A signaling pathway**	38	3	8.21E-03
**APRIL and BAFF signaling pathway**	38	3	8.21E-03

### Synergistic growth inhibition in three neuroblastoma cell lines treated with topotecan and NF-κB inhibitors

**Figure 2:**
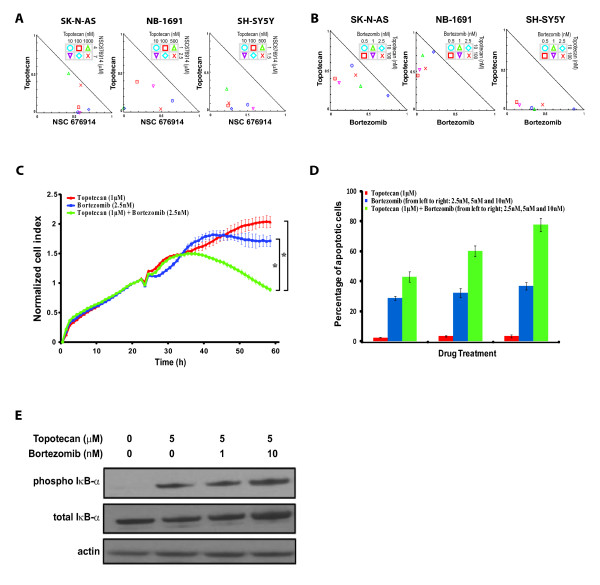
**NF-κB pathway inhibitors potentiated topotecan-induced growth inhibition and caspase-3 activation in neuroblastoma cells**. (**A**) Synergistic growth inhibition effect of topotecan and NSC 676914 in combination in three neuroblastoma cell lines (combination index <1). Sub-IC50 drug doses are used in combination and cell growth is plotted in the normalized isobolograms. (**B**) Normalized isobolograms showed synergy in growth inhibition by the combination of topotecan and bortezomib in three neuroblastoma cell lines. Shown is representative of three independent experiments. (**C**) Synergistic inhibitory effect of topotecan (1 μM) and bortezomib (2.5 nM) on cell proliferation in SK-N-AS cells using a real-time cell electronic sensing system (*P = 0.00005). (**D**) Increased percentage of apoptotic SK-N-AS cells measured by caspase-3 activation with the drug combinations comparing to single drug. (**E**) Immunoblots showed inactivation of the NF-κB pathway by treating cells with bortezomib through inhibition of degradation of total IκB-α protein

In order to examine if the cell growth inhibition in neuroblastoma mediated by topotecan can be synergized with NF-κB pathway inhibition, we tested a specific NF-κB inhibitor, NSC 676914 [19] and a FDA approved proteasome inhibitor which also inhibits the NF-κB pathway, bortezomib, in combination with topotecan. We tested these combinations in SK-N-AS and two additional neuroblastoma cell lines NB-1691 (*MYCN *amplified) and SH-SY5Y (MYCN non-amplified) to ensure that the synergistic effect was not specific to any cell line. We first measured the IC50 of topotecan and NSC676914 in these cells at 24 hours, and they were 2 μM and 7 μM for SK-N-AS; 550 nM and 4 μM for NB-1691; 200 nM and 2 μM for SH-SY5Y respectively. Sub-IC50 doses of topotecan and NSC 676914 were then used in combination in all three cell lines and the combination index was calculated for each dose combination [18]. All the combination treatment data points in normalized isobolograms were away from the diagonal additive line and towards the origin indicating that low doses of NSC676914 and topotecan act synergistically in all three cell lines (Figure 2A). To facilitate future translation of our findings to the clinic, we tested bortezomib, an FDA-approved drug known to inhibit NF-κB as one of its mechanisms, in the subsequent *in vitro *and *in vivo *studies. In addition, this drug has been reported to inhibit all of the top four pathways identified in our synthetic lethal screen (Table 2) [20-22]. Therefore, we predicted bortezomib would have synergistic effects in combination with topotecan in neuroblastoma cells. To test this hypothesis, we first measured the IC50 of bortezomib for these three cell lines at 24 hours as 7 nM, 3.5 nM and 3.5 nM for SK-N-AS, NB-1691 and SH-SY5Y respectively. As expected, we found from normalized isobolograms that sub-IC50 doses of topotecan and bortezomib acted synergistically to inhibit cell growth as the data points in normalized isobolograms were also away from the diagonal additive line (Figure 2B). This synergistic inhibitory effect between topotecan and bortezomib was confirmed in an independent experiment using electronic cell sensing to monitor cell growth (P < 0.01) (Figure 2C). Furthermore, we were able to detect an increase in apoptosis with the combination of topotecan and bortezomib as evidenced by elevated caspase-3 activity (Figure 2D). To confirm that bortezomib enhances topotecan-induced growth inhibition through NF-κB pathway, Western blotting was performed to show that SK-N-AS cells treated with both bortezomib and topotecan inhibited the degradation of total IκB-α protein, suggesting that the NF-κB was indeed inactivated (Figure 2E). Taken together, these data demonstrated that topotecan and NF-κB inhibitors could synergistically inhibit growth through inhibition of the NF-κB pathway and induction of apoptosis in neuroblastoma cells.

### Delayed tumor progression in human neuroblastoma xenograft treated with topotecan and bortezomib

**Figure 3:**
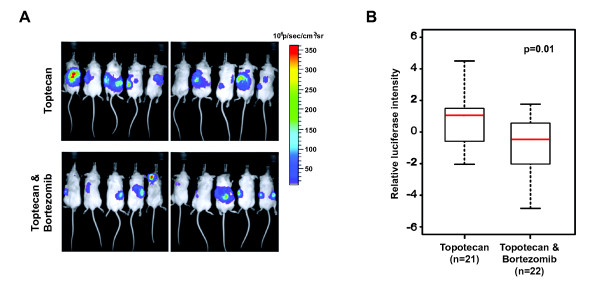
**Delayed tumor progression in human neuroblastoma xenograft treated with topotecan and bortezomib**. Mice were inoculated intravenously with five million SK-N-AS cells expressing luciferase. Seven days later, mice were treated with intraperitoneal injections of topotecan and bortezomib individually or in combination as detailed in methods. **(A) **Representative xenogen images of tumor-bearing mice. Mice receiving the combination of topotecan and bortezomib **(lower panel) **showed decreased tumor burden comparing those treated with topotecan alone **(upper panel)**. Tumor burden was measured by imaging luminescence on a Xenogen IVIS 100 imaging system. **(B) **Quantification of tumor burden demonstrated that mice treated with combination of topotecan and bortezomib (n = 22) had significant less tumor burden than those treated with topotecan only (n = 21) (P = 0.01). This figure represents the results obtained from two independent experiments. We used relative luciferase signals in photons per second of the dorsal view of the two groups at two weeks post treatment. The log2 transformed luciferase intensities from each experiment are normalized using median-centered method and then combined. *T*-test was used to compare the difference of two groups

In order to investigate the synergistic effect of topotecan and bortezomib *in vivo*, we tested the drug combination in SCID beige mice bearing human neuroblastoma xenografts. SK-N-AS cells were intravenously injected into the mice and monitored by Xenogen. Treatments started seven days after injection, and our treatment scheme encompassed only two courses of drug administration allowing us to focus on the immediate effect of the drug combination on tumor formation and progression. One week after the first course of treatment, tumors were observed in mice treated with control and bortezomib alone. By the end of the second course of treatment, these mice became morbid and were euthanized (data not shown). We also found that the mice treated with combination of topotecan and bortezomib showed a delay in tumor progression when compared to mice treated with topotecan alone (Figure 3A). Results from two independent *in vivo *experiments showed that the relative luciferase signals from tumor cells were significantly decreased in mice treated with combination of topotecan and bortezomib (p = 0.01; Figure 3B). Therefore, we concluded that combination therapy of topotecan and bortezomib caused a significant reduction of tumor growth compared to individual drugs alone, which confirmed our hypothesis that bortezomib potentiated the effects of topotecan, via targeting the NF-κB pathway as one mechanism together with the topoisomerase-1 inhibition.

## Discussion

More than half of the neuroblastoma patients over 1 year old have advanced metastatic disease at the time of diagnosis [1]. For these patients, the overall survival rate remains less than 50%. Therefore, a new therapeutic strategy is critically needed. Current treatment regimens used in high risk neuroblastoma include topotecan, a topoisomerase I inhibitor and cyclophosphamide, a nitrogen mustard alkylating agent. Cyclophosphamide induces DNA cross-linking and DNA single-strand breaks; while topotecan inhibits religation of the topoisomerase I-mediated DNA single-strand breaks. Both result in increased numbers of strand breaks and stabilization of these unrepaired breaks, leading to enhanced cytotoxicity. The combination was first proven effective in a phase II trial in neuroblastoma, in which there were six partial responses in 13 patients with neuroblastoma with the combination of cyclophosphamide and topotecan compared with two responses (one complete and one partial) in 37 patients treated with topotecan alone.[11]. Subsequently, topotecan together with other topoisomerase inhibitors have become the basis of many salvage regimens and is being evaluated as up-front therapy in ongoing trials in neuroblastoma and other cancers.

Here we utilized a high throughput loss of function approach using siRNAs to identify genes whose inhibition would synergize with topotecan with the ultimate goal of discovering potent synergistic drug combinations for treating patients with neuroblastoma. SiRNA screening can identify genes, and pathways critical for cancer cell growth and survival. This approach provides a rational method of choosing inhibitors to target the identified genes and pathways. The objective of combination chemotherapy is to simultaneously target multiple pathways that are important for cancer cell growth and survival, in the hope to synergistically inhibit tumor cell growth. In our study, there was an enrichment of NF-κB pathway genes in the positive hits and an induction of NF-κB gene signature upon treatment with topotecan. This pathway plays an important role in inflammation, autoimmune response, cell proliferation, and apoptosis depending on the cell type and context [23]. In cancers including neuroblastoma, the NF-κB pathway is found to activate transcription of genes encoding tumor-promoting cytokines, playing critical roles in neoplastic transformation and cancer cell survival [24]. Hence, targeting this signaling pathway should be effective for growth inhibition and increasing apoptosis for cancers. However, NF-κB inhibitors are generally used as adjuvants because inhibition of NF-κB alone may be insufficient for a pronounced apoptotic response unless it is combined with apoptosis-inducing drugs or radiation [25]. Our results indicated that the NF-κB pathway was activated as a possible protective mechanism against topoisomerase-I inhibition resulting in single stranded DNA breaks. This together with our siRNA screening results suggested that the combination of topotecan and an NF-κB inhibitor might be a good combination to kill neuroblastoma cells.

In this study, we found that a specific NF-κB inhibitor, NSC 676914, synergized growth inhibition mediated by topotecan in both *MYCN *and non-*MYCN *amplified neuroblastoma cell lines. NSC 676914 has been identified as a novel, specific and stable small-molecular NF-κB inhibitor through the inhibition of IKK-β to induce growth inhibition of multiple myeloma cells *in vitro *and *in vivo *[19,26]. Our findings were consistent with a recent publication which suggested that inhibition of NF-κB pathway could lead to increased chemosensitivity [27]. In addition, we used bortezomib, a FDA-approved drug known to have NF-κB inhibition effects through preventing IκB-α protein degradation in the proteasome [28], for the subsequent *in vitro *and *in vivo *studies. Bortezomib, the first FDA-approved proteasome inhibitor, is a boronic-acid derivative that reversibly inhibits the active sites in the 20S proteasome [29,30]. Down-regulation of NF-κB pathway is its prevailing mechanism of action in multiple myeloma and relapsed mantle cell lymphoma [31]. In addition to NF-κB inhibition, bortezomib has been shown to inhibit other pathways which may partially contribute to the effect observed in this study. For example, the topoisomerase I cleavable complex also serves as a substrate for the proteasome [32], an inhibitory target of bortezomib. Stabilization of these complexes has been shown to enhance the cytotoxic effect of another topoisomerase I inhibitor camptothecin, an analog of topotecan [33]. Furthermore, bortezomib inhibits tumor growth, causing cell cycle arrest in colon, ovarian, breast, renal cell and pancreatic carcinomas [34], as well as in lymphomas and leukemias and pediatric tumors including neuroblastoma *in vitro *and *in vivo *[35].

Our *in vivo *xenograft experiments demonstrated a delay in tumor progression when topotecan was combined with bortezomib. Therefore, bortezomib could be a logical choice of NF-κB inhibitors to be used with topotecan as a rational combination therapy. With regards to drug toxicity, myelosuppression, primarily reversible, noncumulative neutropenia, is the predominant toxicity observed with topotecan treatment [11,36]. For bortezomib, while peripheral neuropathy is the most often seen toxicity, recent study suggests that it is manageable and reversible [37]. Bortezomib-induced thrombocytopenia and neutropenia are cyclic, reversible, typically do not lead to treatment discontinuation and recover prior to initiation of the subsequent cycle [38,39]. The combination of topotecan and bortezomib is currently under investigation in clinical trials for other advanced solid tumors in adults [40]. Thus this combination should be well tolerated in patients with neuroblastoma.

## Conclusions

In conclusion, our synthetic lethal siRNA screening led to the discovery that NF-κB inhibition synergized cell death when used with topotecan in neuroblastoma. Furthermore we showed that the NF-κB pathway was induced in neuroblastoma cells treated with topotecan. Finally we demonstrated evidence of the synergistic effects of topotecan and bortezomib in *in-vitro *and in a pre-clinical mouse model. Our study therefore provides the rationale for future clinical trials evaluating this combination therapy for patients with high risk neuroblastoma.

## Competing interests

The authors declare that they have no competing interests.

## Authors' contributions

PT contributed to conception and design, acquisition, interpretation and analysis of the results, and wrote the manuscript. AC contributed to the design of the *in vivo *experiment and carried out a subset of experiments. QC contributed to conception and performed the pathway analysis; gene set enrichment analysis and statistical analyses. YKS contributed to a subset of experiments. TB revised the manuscript. JSW contributed to conception, design, and interpretation of the results for the study; and revised the manuscript. JK devised and directed the study. All authors read and approved the final manuscript.

## Pre-publication history

The pre-publication history for this paper can be accessed here:

http://www.biomedcentral.com/1471-2407/12/101/prepub

## References

[B1] Brodeur GM Maris JM Principles and practice of pediatric oncology 2006 5 Philadelphia: J B Lippincott Company 933 970

[B2] Cecchetto G Surgical risk factors in primary surgery for localized neuroblastoma: the LNESG1 study of the European International Society of Pediatric Oncology Neuroblastoma Group J Clin Oncol 2005 23 33 8483 8489 10.1200/JCO.2005.02.4661 16293878

[B3] Maris JM Neuroblastoma Lancet 2007 369 9579 2106 2120 10.1016/S0140-6736(07)60983-0 17586306

[B4] Gheeya JS Screening a panel of drugs with diverse mechanisms of action yields potential therapeutic agents against neuroblastoma Cancer Biol Ther 2009 8 24 2386 2395 10.4161/cbt.8.24.10184 19946221 PMC2829338

[B5] Matthay KK Treatment of high-risk neuroblastoma with intensive chemotherapy, radiotherapy, autologous bone marrow transplantation, and 13-cis-retinoic acid. Children's Cancer Group N Engl J Med 1999 341 16 1165 1173 10.1056/NEJM199910143411601 10519894

[B6] Gilman AL Phase I study of ch14.18 with granulocyte-macrophage colony-stimulating factor and interleukin-2 in children with neuroblastoma after autologous bone marrow transplantation or stem-cell rescue: a report from the Children's Oncology Group J Clin Oncol 2009 27 1 85 91 19047298 10.1200/JCO.2006.10.3564 PMC2645092

[B7] Frost JD A phase I/IB trial of murine monoclonal anti-GD2 antibody 14.G2a plus interleukin-2 in children with refractory neuroblastoma: a report of the Children's Cancer Group Cancer 1997 80 2 317 333 10.1002/(SICI)1097-0142(19970715)80:2<317::AID-CNCR21>3.0.CO;2-W 9217046

[B8] Kushner BH Pilot study of topotecan and high-dose cyclophosphamide for resistant pediatric solid tumors Med Pediatr Oncol 2000 35 5 468 474 10.1002/1096-911X(20001101)35:5<468::AID-MPO5>3.0.CO;2-P 11070479

[B9] Park JR Pilot induction regimen incorporating pharmacokinetically guided topotecan for treatment of newly diagnosed high-risk neuroblastoma: a Children's Oncology Group study J Clin Oncol 2011 29 33 4351 7 10.1200/JCO.2010.34.3293 22010014 PMC3221519

[B10] London WB Phase II randomized comparison of topotecan plus cyclophosphamide versus topotecan alone in children with recurrent or refractory neuroblastoma: a Children's Oncology Group study J Clin Oncol 2010 28 24 3808 15 10.1200/JCO.2009.27.5016 20660830 PMC2940398

[B11] Saylors RL 3rd Cyclophosphamide plus topotecan in children with recurrent or refractory solid tumors: a Pediatric Oncology Group phase II study J Clin Oncol 2001 19 15 3463 3469 11481351 10.1200/JCO.2001.19.15.3463

[B12] Hertzberg RP Caranfa MJ Hecht SM On the mechanism of topoisomerase I inhibition by camptothecin: evidence for binding to an enzyme-DNA complex Biochemistry 1989 28 11 4629 4638 10.1021/bi00437a018 2548584

[B13] Armstrong DK Hematologic safety and tolerability of topotecan in recurrent ovarian cancer and small cell lung cancer: an integrated analysis Oncologist 2005 10 9 686 694 10.1634/theoncologist.10-9-686 16249347

[B14] Fiorica JV The role of topotecan in the treatment of advanced cervical cancer Gynecol Oncol 2003 90 3 Pt 2 S16 S21 13129491 10.1016/s0090-8258(03)00465-7

[B15] Nitschke R Topotecan in pediatric patients with recurrent and progressive solid tumors: a Pediatric Oncology Group phase II study J Pediatr Hematol Oncol 1998 20 4 315 318 10.1097/00043426-199807000-00006 9703003

[B16] Livak KJ Schmittgen TD Analysis of relative gene expression data using real-time quantitative PCR and the 2(-Delta Delta C(T)) Method Methods 2001 25 4 402 408 10.1006/meth.2001.1262 11846609

[B17] Subramanian A Gene set enrichment analysis: a knowledge-based approach for interpreting genome-wide expression profiles Proc Natl Acad Sci USA 2005 102 43 15545 15550 10.1073/pnas.0506580102 16199517 PMC1239896

[B18] Chou TC Talalay P Quantitative analysis of dose-effect relationships: the combined effects of multiple drugs or enzyme inhibitors Adv Enzyme Regul 1984 22 27 55 6382953 10.1016/0065-2571(84)90007-4

[B19] Kang MI A selective small-molecule nuclear factor-kappaB inhibitor from a high-throughput cell-based assay for "activator protein-1 hits" Mol Cancer Ther 2009 8 3 571 581 10.1158/1535-7163.MCT-08-0811 19258426 PMC2813146

[B20] Demarchi F Brancolini C Altering protein turnover in tumor cells: new opportunities for anti-cancer therapies Drug Resist Updat 2005 8 6 359 368 10.1016/j.drup.2005.12.001 16406769

[B21] Carew JS Giles FJ Nawrocki ST Histone deacetylase inhibitors: mechanisms of cell death and promise in combination cancer therapy Cancer Lett 2008 269 1 7 17 10.1016/j.canlet.2008.03.037 18462867

[B22] Li W New targets of PS-341: BAFF and APRIL Med Oncol 2010 27 2 439 445 10.1007/s12032-009-9230-z 19452288

[B23] Ghosh S Karin M Missing pieces in the NF-kappaB puzzle Cell 2002 109 Suppl S81 S96 11983155 10.1016/s0092-8674(02)00703-1

[B24] Small MB Neoplastic transformation by the human gene N-myc Mol Cell Biol 1987 7 5 1638 1645 3299052 10.1128/mcb.7.5.1638-1645.1987 PMC365263

[B25] Karin M Nuclear factor-kappaB in cancer development and progression Nature 2006 441 7092 431 436 10.1038/nature04870 16724054

[B26] Hideshima T MLN120B, a novel IkappaB kinase beta inhibitor, blocks multiple myeloma cell growth in vitro and in vivo Clin Cancer Res 2006 12 19 5887 5894 10.1158/1078-0432.CCR-05-2501 17020997

[B27] Amschler K et al NF-kappaB inhibition through proteasome inhibition or IKKbeta blockade increases the susceptibility of melanoma cells to cytostatic treatment through distinct pathways J Invest Dermatol 2010 130 4 1073 86 10.1038/jid.2009.365 19940859

[B28] Sartore-Bianchi A Bortezomib inhibits nuclear factor-kappaB dependent survival and has potent in vivo activity in mesothelioma Clin Cancer Res 2007 13 19 5942 5951 10.1158/1078-0432.CCR-07-0536 17908991

[B29] Nencioni A Proteasome inhibitors: antitumor effects and beyond Leukemia 2007 21 1 30 36 10.1038/sj.leu.2404444 17096016

[B30] Richardson PG Bortezomib: proteasome inhibition as an effective anticancer therapy Annu Rev Med 2006 57 33 47 10.1146/annurev.med.57.042905.122625 16409135

[B31] Orlowski RZ Kuhn DJ Proteasome inhibitors in cancer therapy: lessons from the first decade Clin Cancer Res 2008 14 6 1649 1657 10.1158/1078-0432.CCR-07-2218 18347166

[B32] Desai SD Ubiquitin-dependent destruction of topoisomerase I is stimulated by the antitumor drug camptothecin J Biol Chem 1997 272 39 24159 24164 10.1074/jbc.272.39.24159 9305865

[B33] Cusack JC Jr Enhanced chemosensitivity to CPT-11 with proteasome inhibitor PS-341: implications for systemic nuclear factor-kappaB inhibition Cancer Res 2001 61 9 3535 3540 11325813

[B34] Adams J Development of the proteasome inhibitor PS-341 Oncologist 2002 7 1 9 16 10.1634/theoncologist.7-1-9 11854543

[B35] Brignole C Effect of bortezomib on human neuroblastoma cell growth, apoptosis, and angiogenesis J Natl Cancer Inst 2006 98 16 1142 1157 10.1093/jnci/djj309 16912267

[B36] Bence AK Adams VR Adams VR, Burke TG Clinical Experience With Topotecan Camptotehcins in cancer therapy 2005 Humana Press Inc, Totowa, NJ 268

[B37] Richardson PG Reversibility of symptomatic peripheral neuropathy with bortezomib in the phase III APEX trial in relapsed multiple myeloma: impact of a dose-modification guideline Br J Haematol 2009 144 6 895 903 10.1111/j.1365-2141.2008.07573.x 19170677

[B38] Moehler T Goldschmidt H Therapy of Relapsed and Refractory Multiple Myeloma Multiple Myeloma 2011 Springer-Verlag Berlin Heidelberg, Germany 252 10.1007/978-3-540-85772-3_11 21509688

[B39] Velcade Prescribing information http://www.millennium.com/pdf/VelcadePrescribingInformation.pdf

[B40] Lara PN Jr Bortezomib (PS-341) in relapsed or refractory extensive stage small cell lung cancer: a Southwest Oncology Group phase II trial (S0327) J Thorac Oncol 2006 1 9 996 1001 10.1097/01243894-200611000-00013 17409985

